# Endoscopy-assisted medial canthus incision for olfactory neuroblastoma: a case report

**DOI:** 10.1186/s12957-024-03448-9

**Published:** 2024-06-22

**Authors:** Yanwen Li, Xin Zhao, Yanli Tao

**Affiliations:** 1 School of Clinical Medicine, Shandong Second Medical University, Weifang, China; 2 Weifang People’s Hospital, No. 151 Guangwen Street, Kuiwen District, Wei- fang, 261041 Shandong China

**Keywords:** Endoscopy, Sinonasal malignant tumors, Olfactory neuroblastoma

## Abstract

Sinonasal malignant tumors are a group of uncommon malignancies that account for less than 1% of all tumors. These tumors often involve the maxillary sinus and nasal cavity, with less cumulative incidence in the ethmoidal sinus, sphenoidal sinus, and frontal sinus. The lack of consensus on the management of sinonasal malignancies is due to their rarity, diagnostic challenges, and the heterogeneity of treatments. In this paper, we present a case of endoscopic-assisted medial canthus incision combined with radiotherapy in the treatment of sinonasal malignant tumors, with the aim of providing valuable insights to clinicians on the management of these tumors.

## Background

A case study of an elderly female patient who was diagnosed with left ethmoidinal neuroblastoma is presented in this article. The patient underwent radiotherapy after intranasal endoscopy assisted medial canthal incision. Postoperative follow-up showed that the patient had recovered well after surgery, and smooth mucosa in the left ethmoidal surgery area was observed under postoperative nasal endoscopy, without any abnormal raised mucosa. Intranasal endoscopy assisted medial canthus incision is a new multimodal treatment of ethmoid malignant tumors that has shown promising results in terms of oncology outcomes. This treatment approach is an alternative to open conventional craniofacial resection and has been found to be equally effective.

Surgical resection followed by adequate surgical adjuvant radiotherapy, combined with or without chemotherapy, is the most acceptable treatment for olfactory neuroblastoma. The use of intranasal endoscopy assisted medial canthus incision in this multimodal treatment approach has been found to be a safe and effective alternative to open conventional craniofacial resection.

## Case report

**Fig. 1:**
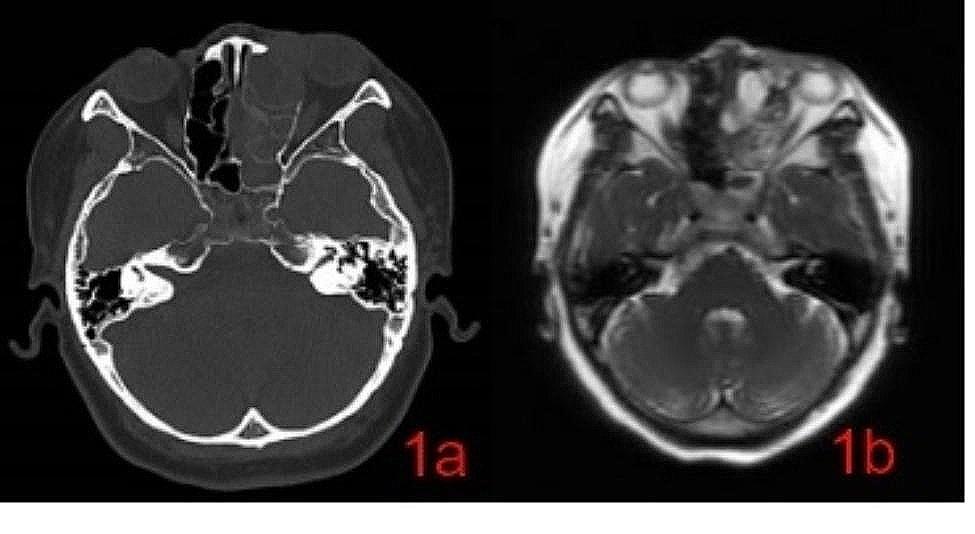
**a**: CT: The presence of erosion in the left sinus wall, the cricoid plate, and the left intraorbital wall. **b**: MRI: The mean signal of the mass on the t2-weighted sequence was high signal, with unclear boundaries and large invasion range

A female patient aged 53 years reported with a persistent left nasal congestion of 2 years duration, accompanied by purulent discharge, anosmia, dull left head pain, recurrent epiphora in the left eye, photo-phobia, blurred vision, itchy eyes, periorbital pain, and left upper denopain symptoms. The patient denied any history of smoking or alcohol consumption and did not have any known genetic predisposition to her condition. Upon examination, it was noted that the septum had deviated towards the right side. Additionally, there was evidence of polypoid neobiology in the left middle nasal passage, and noticeable tenderness was observed in the left periodontal region. The remaining of physical examination and laboratory examination showed no obvious abnormalities. The report of Plain CT scan and MRI highlights the left nasal cavity, frontal sinus, ethmoid sinus, and maxillary sinus are occupying space and the presence of inflammation in the left nasal cavity, frontal sinus, ethmoid sinus, and maxillary sinus, indicating a condition of paranormal sinus inflammation. In addition, the report also mentions the existence of a submucosal cyst in the right maxillary sinus and nasal septal deviation. (Figure 1a and b). The patient was administered general anesthesia for a downward endoscopic left group of sinus fenestration and endoscopic ethmoid sinus malignant tumor resection. Intraoperatively, the medical team observed a smooth mass with transparent secretion in the left nasal cavity, as well as maxillary sinus atresia, maxillary sinus sieve, sphenoid sinus, and frontal sinus that were full of white purulent secretions. The tumor was found to be closely adhered to the orbital periosteum, and there was a significant defect in the orbital cardboard. The postoperative pathology report of the patient revealed the presence of olfactory neuroblastoma, grade 2, which was confirmed using immunohistochemistry. The immunohistochemical analysis showed that the tumor cells expressed CD56, Syn, NSE, and S-100 (supporting cell +), while they were negative for Vimentin, HMB 45, Desmin, P40, CK5 / 6, and NUT. The expression of CK was wide, with little positivity, and CD99 showed little positivity as well. The SOX 10 marker was minimally expressed, and the Ki-67 index was determined to be 2%. The patient received radiotherapy with a total dose of 54 Gy, and 1-year postoperative follow-up revealed no recurrence of the tumor. Furthermore, the patient experienced a significant improvement in symptoms such as nasal congestion.

**Fig. 2:**
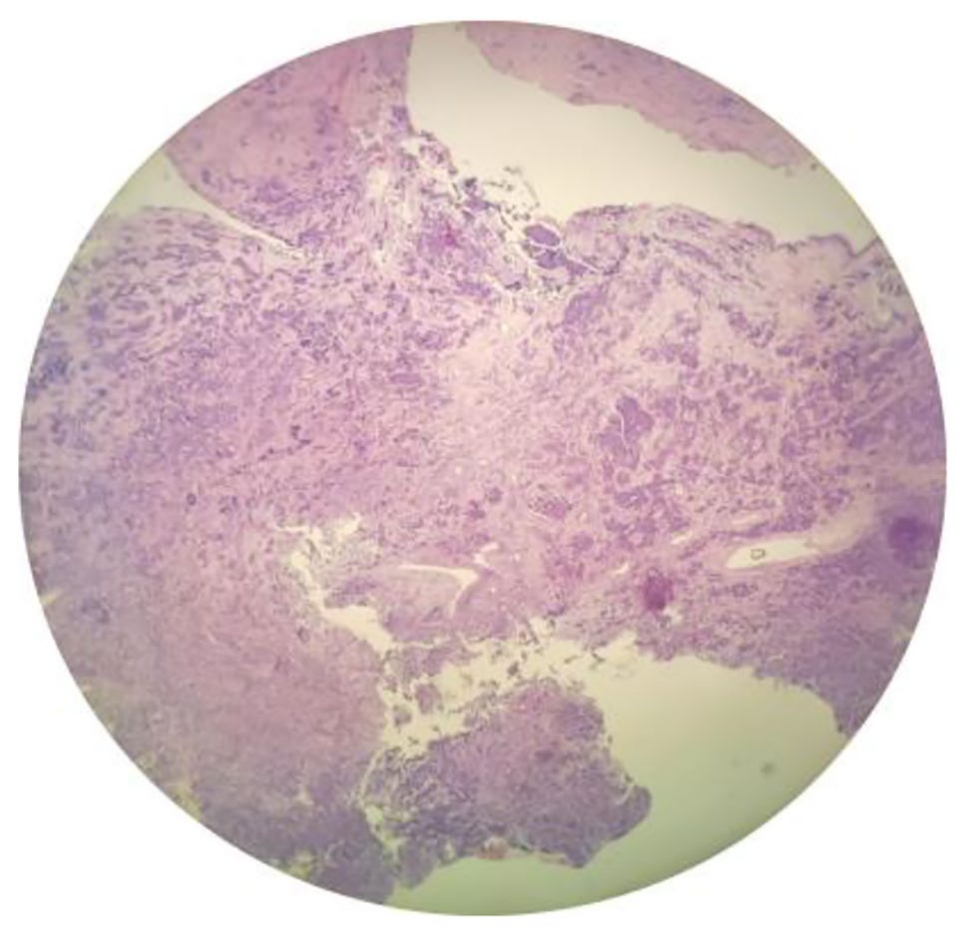
Pathological examination (H&E. 40×): Olfactory neuroblastoma which is high nucleo-cytoplasmic ratio, deep round nuclei, inconspicuous nucleoli, scarce cytoplasm, and abundant intercellular vascularized fibromyxoid stroma

## Discussion

Olfactory neuroblastoma (ONB) is a rare malignant tumor that is primarily restricted to the olfactory nerve epithelium in the nasal sinus. Despite several studies, the etiology of this type of tumor is not yet fully understood. ONB affects individuals of all genders, with a higher incidence among people aged 11 to 20 years and 51 to 60 years. The tumor mainly develops in the nasal cavity, with the ethmoid sinus, anterior skull base, and orbit being the most common sites. Clinical manifestations of ONB include nasal congestion, olfactory loss, repeated epistaxis, headache, and eye discomfort, such as tears, double vision, and vision loss [1].

The diagnosis of ONB requires a thorough initial examination of nasal and sinonasal malignancies, which should include a physical examination, including neurological assessment, and imaging examinations [2].Typically, ONB presents as a red polypoid mass located at the top of the nasal cavity and middle nasal tract, while the CT reveals a uniform soft tissue mass with a necrotic non-enhanced area. Additionally, a typical imaging feature of ONB is a dumbbell mass through the sieve plate. The MRI shows a low signal in T1-weighted sequences and a mid-to-high signal in T2-weighted sequences [3].The CT and MRI imaging studies of a patient with ONB revealed the presence of filling soft tissue density shadow in the left nasal cavity, frontal sinus, ethmoid sinus, maxillary sinus, local density to left orbit and facial subcutaneous lesion, adjacent bone cortex thinning, local choid sinus wall bone discontinuous, left maxillary sinus cavity compression smaller, left hook process shape unstructured, left sieve funnel occlusion. The MRI scan further showed slightly short T1 long T2 signal shadow in the left nasal cavity and frontal sinus, along with a visible, short T2 signal shadow and fluid level, and unstructured lesion shape with local diffusion limitation. The findings suggest that physical examination and imaging studies are critical for assessing the severity of the condition in the diagnosis and treatment of this rare malignancy, ONB [4]. These results may aid in the development of effective treatment plans for patients with ONB.

The diagnosis of ONB is typically confirmed using pathological examination, which is considered the gold standard for diagnosis. ONB is characterized as a blue small round cell tumor located in the nasal cavity and sinuses, which is comprised of cells with a small round nucleus and punctuate chromatin, arranged in nests or sheets by a neurofibrillary matrix that exhibits classical Flexner-Wintersteiner and Homer Wright rosettes [5]. ONB originates from the upper nasal neuroectoderm and displays neuroendocrine histologist characteristicsI.The immunohistochemical staining of ONB is usually positive for neuroendocrine markers such as synaptophysin, chromogranin, and neuron-specific enolas [2].Therefore, undifferentiated carcinoma should be ruled out through pathological and immunohistochemical identification before making a diagnosis.

ONB is a rare malignancy for which the optimal treatment strategy remains controversial. However, multi-mode combination therapy is generally recommended. The latest treatment approach involves endoscopic surgery and reconstruction combined with local radiotherapy, although the role of neoadjuvant chemotherapy is yet to be clearly defined [6]. Based on the Kadish stage and Hyams grade classification system [7], the patient was in stage Kadish B grade 2, using endoscopic-assisted medial canthal incision instead of open conventional craniofacial resection combined with local radiotherapy for the management of ethmoidal sinus neuroblastoma. During the procedure, a straightforward intranasal approach was used with local anesthesia and an arc incision at the edge of the left nasal bone. This method allowed full exposure of the sinus and the left orbital periosteal lesions. The remaining tumor tissue was resected using 40 degrees bite forceps while ensuring that the orbital fat remained intact. The orbital periosteal and lateral wall of the nasal cavity were observed with a 70-degree endoscope which showed no indication of any remaining tumor tissue. This approach provides a secure and effective option for treating similar cases.According to a retrospective study by Ozsahin et al. [1] the combination of surgery and postoperative radiotherapy has been shown to have the best effect and the highest 5-year survival rate, particularly when the total dose of radiotherapy administered is 54 Gy.In addition, the use of postoperative radiotherapy may decrease the chance of local tumor recurrence and improve local control rates.As part of the treatment, the patient received a dose of 54 Gy. Currently, postoperative review shows no signs of recurrence.

## Conclusion

ONB, a malignancy originating from the nasal and para-nasal sinuses, is known for exhibiting slow growth, high invasiveness, and a proclivity for both local and distant metastasis. Endoscopic surgery and reconstruction, accompanied by local radiotherapy, have been identified as the most effective treatment modalities. However, postoperative recurrence is a potential concern due to the aforementioned biological characteristics of ONB, which underscores the importance of long-term follow-up as an indispensable component of the treatment plan.

## Data Availability

No datasets were generated or analysed during the current study.

## Abbreviations

ONB: Olfactory neuroblastoma
